# Anti-Inflammatory and Anti-Rheumatic Potential of Selective Plant Compounds by Targeting TLR-4/AP-1 Signaling: A Comprehensive Molecular Docking and Simulation Approaches

**DOI:** 10.3390/molecules27134319

**Published:** 2022-07-05

**Authors:** Ashrafullah Khan, Shafi Ullah Khan, Adnan Khan, Bushra Shal, Sabih Ur Rehman, Shaheed Ur Rehman, Thet Thet Htar, Salman Khan, Sirajudheen Anwar, Ahmed Alafnan, Kannan RR Rengasamy

**Affiliations:** 1 Pharmacological Sciences Research Lab, Department of Pharmacy, Faculty of Biological Sciences, Quaid-i-Azam University, Islamabad 45320, Pakistan; ashrafwazir6@gmail.com (A.K.); adkhan165sbbu@gmail.com (A.K.); bushra.shal@gmail.com (B.S.); 2 Faculty of Pharmaceutical Sciences, Abasyn University, Peshawar 25000, Pakistan; shafiullahpharmd@gmail.com; 3 Product & Process Innovation Department, Qarshi Brands (Pvt) Ltd., Hattar 22610, Pakistan; 4 Faculty of Health Sciences, IQRA University, Islamabad Campus (Chak Shahzad), Park link Rd., Islamabad 44000, Pakistan; 5 Department of Pharmacy, Forman Christian College (A Chartered University), Lahore 54600, Pakistan; sabikhan19@gmail.com (S.U.R.); dr.shaheedmarwat@yahoo.com (S.U.R.); 6 School of Pharmacy, Monash University Malaysia, Jalan Lagoon Selatan, Bandar Sunway, Subang Jaya 47500, Selangor, Malaysia; thet.thet.htar@monash.edu; 7 Department of Pharmacology and Toxicology, College of Pharmacy, University of Hail, Hail 55211, Saudi Arabia; si.anwar@uoh.edu.sa (S.A.); a.alafnan@uoh.edu.sa (A.A.); 8 Center of Excellence for Pharmaceutical Sciences, North-West University, Potchefstroom 2520, South Africa; 9 Center for Transdisciplinary Research, Department of Pharmacology, Saveetha Institute of Medical and Technical Sciences (SIMATS), Saveetha Dental College, Chennai 600077, India

**Keywords:** natural products, TLR-4, NF-κB, AP-1, inflammation

## Abstract

Plants are an important source of drug development and numerous plant derived molecules have been used in clinical practice for the ailment of various diseases. The Toll-like receptor-4 (TLR-4) signaling pathway plays a crucial role in inflammation including rheumatoid arthritis. The TLR-4 binds with pro-inflammatory ligands such as lipopolysaccharide (LPS) to induce the downstream signaling mechanism such as nuclear factor κappa B (NF-κB) and mitogen activated protein kinases (MAPKs). This signaling activation leads to the onset of various diseases including inflammation. In the present study, 22 natural compounds were studied against TLR-4/AP-1 signaling, which is implicated in the inflammatory process using a computational approach. These compounds belong to various classes such as methylxanthine, sesquiterpene lactone, alkaloid, flavone glycosides, lignan, phenolic acid, etc. The compounds exhibited different binding affinities with the TLR-4, JNK, NF-κB, and AP-1 protein due to the formation of multiple hydrophilic and hydrophobic interactions. With TLR-4, rutin had the highest binding energy (−10.4 kcal/mol), poncirin had the highest binding energy (−9.4 kcal/mol) with NF-κB and JNK (−9.5 kcal/mol), respectively, and icariin had the highest binding affinity (−9.1 kcal/mol) with the AP-1 protein. The root means square deviation (RMSD), root mean square fraction (RMSF), and radius of gyration (RoG) for 150 ns were calculated using molecular dynamic simulation (MD simulation) based on rutin’s greatest binding energy with TLR-4. The RMSD, RMSF, and RoG were all within acceptable limits in the MD simulation, and the complex remained stable for 150 ns. Furthermore, these compounds were assessed for the potential toxic effect on various organs such as the liver, heart, genotoxicity, and oral maximum toxic dose. Moreover, the blood–brain barrier permeability and intestinal absorption were also predicted using SwissADME software (Lausanne, Switzerland). These compounds exhibited promising physico-chemical as well as drug-likeness properties. Consequently, these selected compounds portray promising anti-inflammatory and drug-likeness properties.

## 1. Introduction

Inflammation is the critical event involved in the pathophysiology of numerous diseases [1] and is considered as a protective response of the body toward the invading agent, however, if not managed properly, then it became troublesome [1,2]. The inflammatory process involves the immune system, vascular compartments, and inflammatory mediators that decide the fate of ongoing inflammatory process [3]. The immune cell infiltration involves neutrophils, macrophages, and their products such as cytokines and chemokines with the ambition to fend off the inflammatory process [4]. However, these mediators leak into systemic circulation and produce generalized effects such as pain, fever, and discomfort. The inflammatory cytokines are the key player involved in the dissemination of local effects into a generalized effect on the body. The various inflammatory mediators that are involved in inflammation include interleukin-1β (IL-1β), interleukin-6 (IL-6), and tumor necrosis factor-α (TNF-α). The immune cells release these inflammatory mediators to facilitate inflammatory events including the infiltration of the immune cells and the dilation of the local vessels. However, the infiltrated immune cells further release the immune cells via a positive feedback mechanism, which leads to the further aggravation of the inflammatory process [5].

The inflammatory cytokine production and release is facilitated by the upstream signaling mechanism such as TLR-4/AP-1 [6]. The Toll-like receptor-4 (TLR-4) binds with the LPS and triggers the activation of the downstream signaling such as nuclear factor κappa B (NF-κB) and mitogen activated protein kinases (MAPKs) [6]. The LPS activation induced the phosphorylation of the MyD88 protein, TIR Domain Containing Adaptor Protein (TIRAP), and the TIR-domain containing adapter-inducing interferon-β (TRIF) protein, which are linked with the downstream MAPKs (JNK) and NF-κB [7]. The NF-κB is inactive within the cytosol under the influence of the inhibitor of nuclear factor kappa B (IκB), however, any inflammatory insult facilitates the phosphorylation and subsequent degradation of IκB [7]. Following degradation of IκB, the NF-κB (p65 and p50) becomes free and translocated to the nucleus to influence the expression of the concerned genes associated with the inflammation and oxidative stress [8]. Similarly, the activation of MAPKs is facilitated by the LPS interaction with the TLR-4, which induces the expression of the concerned genes by AP-1 (activated protein-1) signaling. The AP-1 is the transcriptional factor, which regulates the expression of the numerous genes associated with the inflammation, oxidative stress, and cell growth [8].

Natural products are a rich and cheap source of in the development of new drugs and several plant-derived compounds are in clinical practice for various remedies [9]. The natural product-derived compounds and semi-synthetic compounds provide an important source of a new class of compounds [10]. Similarly, the computational approach has recently been focused on by several researchers, which serves as a reliable and rapid method of drug screening against various diseases. The molecular docking provides the binding affinity of the compounds with the protein target and the mechanism by which this interaction occurs [10]. The computational approach is considered as a reliable method for screening larger number of compounds for their biological activities and the mechanism with which the ligands bind with the protein target [11,12]. In the present study, the computational approach was used to assess the anti-inflammatory activity of the selected compound from natural sources and to explore the possible mechanism of interaction. Furthermore, the computational approach was used to assess the various biological properties such as the pharmacokinetic, physico-chemical, and toxicokinetic behavior of the studied compounds

## 2. Methods

### 2.1. Protein Selection

In the present study, four proteins were selected as targets expressed in inflammation for docking and downloaded as a PDB file from the protein data bank including TLR-4 (4g8e), NF-κB (1le5), JNK (30xi), and AP-1 (4hmy) [13,14]. Swiss PDB viewer software was used for the energy minimization of target proteins. The TLR-4 is a surface protein and LPS interacts as a ligand, which recruits the downstream proteins such as MyD88 toward the cytoplasmic membrane [15]. The MyD88 is an adaptor protein along with the TIRAP and TRIP recruited to the plasma membrane following TLR-4 activation [16]. The MyD88 induces the MAPKs (JNK) and NF-κB signaling, which interacts with the AP-1 signaling to induce the expression of the concerned genes [17].

### 2.2. Ligand Database Preparation

**Figure 1:**
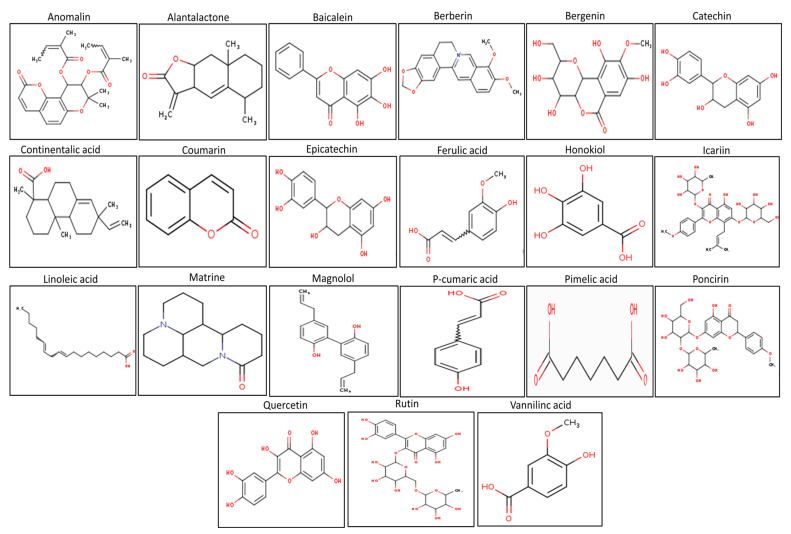
The selected plant product docked against the TLR-4, NF-κB, JNK, and AP-1.

In the present study, 22 plant compounds (Figure 1) were selected based on their reported anti-inflammatory activities [18]. In earlier investigations (liver injury, lung injury, inflammatory pain, colitis, etc.), the selected compounds showed considerable anti-inflammatory activity and marked improvement in the underlying inflammatory activities. These compounds, on the other hand, were not tested for anti-arthritic activity against TLR-4 and downstream signaling proteins such as MAPKs (JNK), AP-1 (activation protein-1), and NF-κB [19,20]. TLR-4 signaling plays a key role in arthritis pathogenesis by activating a number of downstream signaling proteins including MAPKs (JNK), AP-1, and NF-κB. The activation of these signaling pathways results in the production of pro-inflammatory cytokines, aggravating the joint’s inflammatory milieu [20,21]. Furthermore, despite their different biological functions, the pharmacokinetic and toxicokinetic aspects of these drugs have not been thoroughly investigated. Any compound’s pharmacokinetic and toxicokinetic properties have a substantial impact on its pharmacodynamic efficacy and ability to be used clinically [19,20,21]. These compounds were retrieved from the PubChem database and downloaded as a three-dimensional (3D) representation in the SDF structural format (SDF) [22]. The ligands were converted to the PDB file using Open Babel software and their energies were minimized [23].

### 2.3. Docking Protocol

The downloaded protein structures were downloaded in the PDB format and MGL Tools was used to convert them into the pre-requisite PDBQT format [24]. The proteins were prepared by adding polar hydrogen atoms, and gasteiger charges before docking were processed in MGL Tools. The AutoDock Vina software was used to perform the docking analysis [25]. The size and dimension of the Grid box was selected using co-crystalized ligand coordinates within the target protein [26]. Visualization of the protein–ligand interaction was performed using Discovery Studio Visualizer_16 for the presentation in two- and three-dimensions [27].

### 2.4. Pharmacokinetics Parameters Assessment

The pharmacokinetic behaviors of the selected compounds were assessed using SwissADME and pkCSM software [28,29]. The various parameters that were considered included intestinal absorption, the blood–brain barrier (BBB), Caco-2 cell, metabolism, and P-glycoprotein substrate [28,30].

### 2.5. Toxicokinetic Parameter Assessment

The toxicokinetic parameters were assessed with pkCSM and SwissADME using the computational approach [27]. The toxicity of the selected compounds was assessed for minnow toxicity, hepatotoxicity, skin sensitivity, cardiac toxicity, and oral maximum tolerable dose [31].

### 2.6. Drug-Likeness Analysis

The drug-likeness behavior of the studied compounds was analyzed using the bioinformatics software SwissADME and pkCSM [32]. The drug likeness behavior provides a qualitative insight into the pharmacokinetic parameters of the compounds after oral administration using the Lipinski rule including their absorption, bioavailability, and physico-chemical characteristics [33]. Similarly, the toxicokinetic profile of the studied compounds was investigated computationally against the liver, heart, and Ames toxicity test [34].

### 2.7. MD Simulation

Molecular dynamic simulation was performed using YASARA structure software (version 14.12.2) (Wien, Austria) by selecting the AMBER14 force field [35]. The whole protein was embedded in the water-filled simulation cell (20 Å). During the entire simulation process, the experimental condition was maintained at a constant pressure (107p) and a temperature of 298 K. The TLR-4 protein and rutin molecule was placed at the center of the cubic box, adding the counterion to adjust the pH to the physiological level (i.e., 7.4) [36]. The simulation was commenced for 150 ns with a 2.5 fs time step at a constant temperature and pressure (NPT ensemble) [37]. A pre-established macro script (md run.mcr) within the YASARA package was used during the simulation steps and the output of the conformation was 100 ps [38]. The results of the simulations were visualized using Discovery Studio Visualizer version 2018.

### 2.8. Analysis of MD Simulation and Calculation of Secondary Structure Content

The TLR-4 protein flexibility was analyzed during the simulation process by assessing the different parameter of all of the amino acid residues for 150 ns [39,40]. The RMSD and RMSF, which indicate the overall stability of the hit compounds, were calculated. The various factors that were assessed included C alpha, backbone, and the RMSD of all of the atoms of the TLR-4 and rutin complex for 150 ns [39,41]. The secondary structural features of the TLR-4 protein before the MD simulation and after the simulation were assessed using secondary YASARA software. The results also consist of the percent contribution of the sheet, helix, turn, and coil compared to the total structure composition [39,42].

### 2.9. Statistical Analysis

The GraphPad Prism version 5 software (GraphPad software, San Diego, CA, USA) was used to plot the docking score of the protein and ligand complex.

## 3. Results

### 3.1. The Physico-Chemical Analysis of Selected Compounds

The physico-chemical analysis of the selected compounds was assessed using a computational tool (swissAMDE software) [43]. The various properties that were evaluated included molecular weight, LogP, the number of rotational bonds, the number of H-bond acceptors, the number of H-bond donors, and surface area [43]. The compounds showed variable physico-chemical properties, as shown in Table 1. Icariin showed higher molecular weight, while the linoleic acid showed the lowest molecular weight. Rutin showed the lowest LogP value, while linoleic acid exhibited the highest LogP value. The linoleic acid showed the highest rotational bonds, while the matrine, coumarin, and alactolactone revealed the lowest rotational bonds. Similarly, icariin showed the highest H-bond acceptors, while linoleic acid and continentalic acid showed the lowest H-bond acceptors. Rutin showed the highest H-bond donor value, while anomalin, alantolactone, matrine, coumarin, and berberin exhibited a lower H-bond energy. Icariin showed the maximum surface area, while coumarin showed the lowest surface area, as shown in Table 1.

### 3.2. Toxicokinetic Analysis of Selected Natural Compounds

Except for berberin, no compound showed Ames toxicity, while no compound showed herG I toxicity and only honokiol, icariin, magnolol, poncirin, and rutin showed herG II toxicity, as shown in Table 2. Similarly, anomalin, berberin, continentalic acid and linoleic acid showed hepatotoxicity, while alantolactone, linoleic acid, and matrine showed skin sensitivity. Furthermore, the compounds showed variable properties with respect to T. Pyriformis toxicity, minnow toxicity, and oral toxicity, as shown in Table 2.

### 3.3. Pharmacokinetic Behavior

The pharmacokinetic parameters of the selected compounds were evaluated using SwissADME software [44]. The pharmacokinetic parameters that were studied included GIT absorption, blood–brain barrier, Caco-2 cell permeability, glycoprotein substrate and inhibitor, and cytochrome p450 isoenzyme inhibition [32]. The selected compounds showed diverse pharmacokinetic behavior with respect to absorption, blood–brain barrier penetration, Caco-2 cell permeability, glycoprotein (serving as the substrate and inhibitor of the glycoprotein), and cytochrome p450 isoenzyme inhibition, as shown in Table 3.

### 3.4. Drug-Likeness Behavior of the Studied Compounds

The drug-likeness properties such as the Lipinski rule, Ghose, Veber, Egan, and Muegge rule can be found using the computational approach [45]. Most of the study compounds followed the Lipinski rule except for icariin, poncirin, and vanillic acid. However, the compounds showed variable properties in terms of the Ghose, Veber, Egan, and Muegge rule, as shown in the Table 4.

### 3.5. Bioavailability Radar Analysis of the Drugs

**Figure 2:**
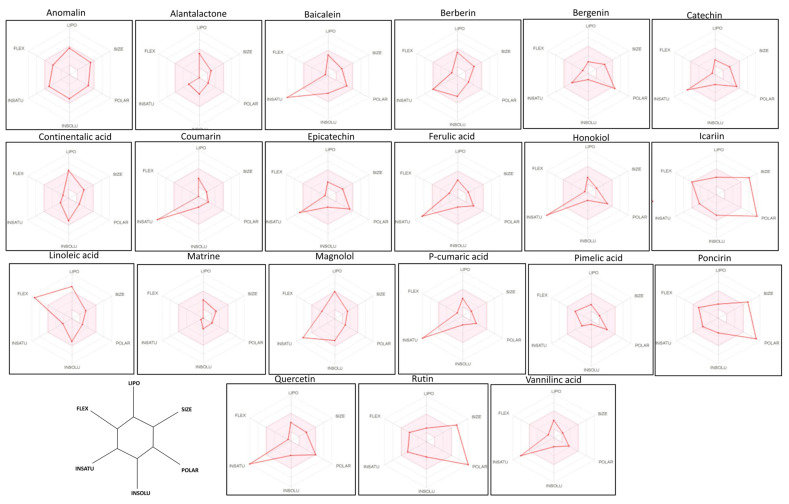
The bioavailability radar analysis of the selected compounds using SwissADME software. The various properties that were assessed included LIPO, FLEX, SIZE, POLAR, INSOLU, and INSATU, which indicates the lipophilicity, Molecular weight, polarity, water solubility, etc. The compounds lying within the gray area reflect a good pharmacokinetic and drug profile.

Radar analysis was performed for all of the studied compounds, which is a computational tool that gains ideas about the rule, followed by compounds such as FLEX, LIPO, SIZE, POLAR, INSATU, and INSOLU [45]. These parameters provide an insight into the drug likeness behavior of the studied compounds. The bioavailability radar analysis was analyzed using the computational approach (pink area represent optimal range of particular property) using SwissADME software. The LIPO represents lipophilicity as the XLOGP3, size represents the molecular weight of the compound, POLAR (topological polar surface area) represents the polarity, INSOLU represents the insolubility in water by log S scale, INSATU represents the instauration as per fraction in the carbon in the SP3 hybridization, and FLEX represents the flexibility as per rotatable bonds. The anomalin, matrine, pimelic acid, alantolactone, and bergenin showed promise as a bioavailability radar, as shown in Figure 2.

### 3.6. Boiled Egg Analysis

**Figure 3:**
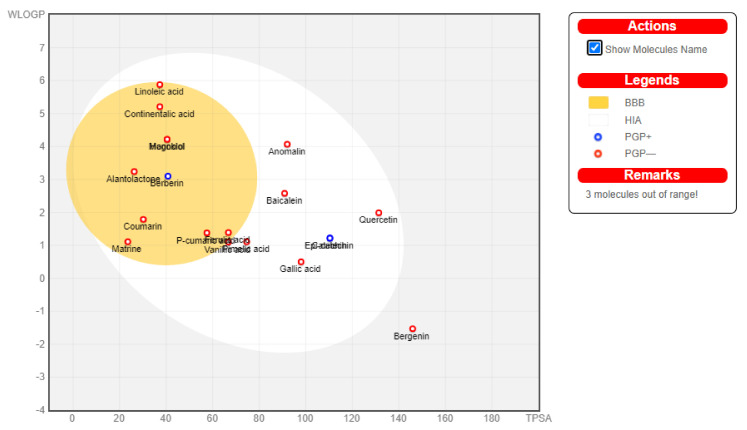
The boiled egg analysis was performed to assess the blood–brain barrier and human intestinal absorption of the studied compounds. The compounds that can penetrate the blood–brain barrier lie within the yellow color, while those lying within the yellow color indicate not permeable to the brain.

The SwissADME software was used for the boiled egg analysis [46]. The boiled egg analysis provides useful information about the BBB, HIA, PG+, and PG- [47]. The compounds that showed BBB permeability included linoleic acid, continentalic acid, magnolol, honokiol, alantolactone, berberine, coumarin, matrine, p-cumaric acid, ferulic acid, and vanillic acid while the rest of the compounds showed intestinal absorption, but no BBB permeability, as shown in Figure 3.

### 3.7. Molecular Docking of Selected Compounds with TLR-4

**Figure 4:**
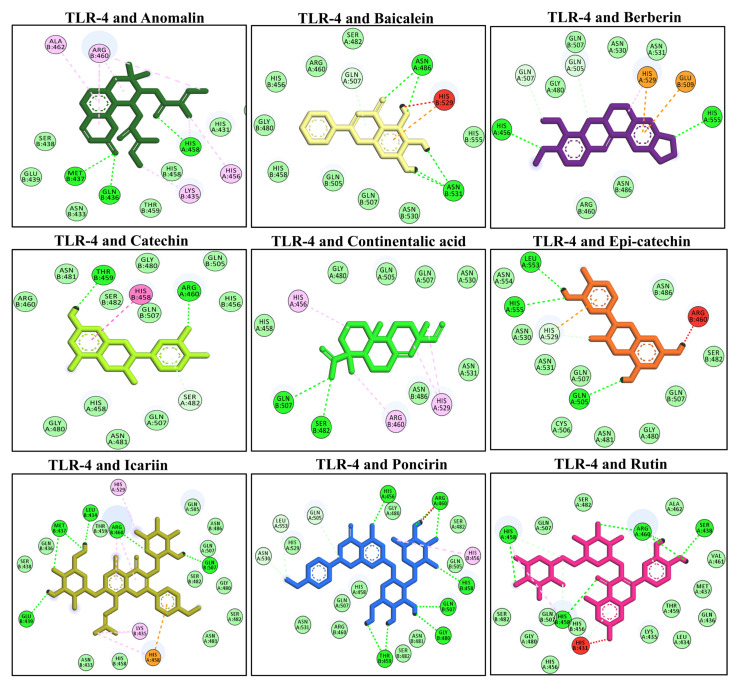
The molecular docking analysis of the selected compounds with the TLR-4 receptor using AutoDock Vina software. The molecular docking showed binding of the studied ligand with the TLR-4 via multiple hydrophilic and hydrophobic bonds. The ligand and protein interactions were visualized in Discovery Studio Visualizer_16 and ligands whose negative binding energy was high were plotted.

The TLR-4 is a surface receptor that interacts with the LPS to induce the downstream signaling and produce the inflammatory response [48]. The molecular docking analysis of the selected compounds was performed using AutoDock Vina software against the TLR-4 receptor. The compounds showed different affinity toward the target protein (i.e., TLR-4). Among these compounds, anomalin, baicalein, berberin, catechin, continentalic acid, epi-catechin, icariin, poncirin, and rutin have shown the lowest binding score. These compounds interacted via multiple hydrophilic and hydrophobic bonds. The anomalin exhibited three H-bonds (Met B437, His A458, Gln B436) with the TLR-4 receptor. Similarly, baicalein binds with the TLR-4 protein via two H-bonds (ASN A486, ASN B531), berberin binds via two H-bonds (His A456, His A555), catechin binds via two H-bonds (Thr B459, ARG A460), continentalic acid binds via two H-bonds (Gln B507, Ser B482), and epi-catechin binds via three H-bonds (Leu A553, His A555, Gln A505) with TLR-4. Moreover, icariin binds with the TLR-4 via five H-bonds (Leu B434, Arg B460, Gln B507, Met B437, Glu B439), poncirin binds by five H-bonds (His A456, Arg A460, His B458, Gln B507, Gly B480, Thr B459), and rutin via four H-bonds (His A458, Arg A460, Ser A438, His B458), as shown in Figure 4. The molecular docking energies are represented in kcal/mol and are presented in Table 5.

### 3.8. Docking Interaction with the NF-κB

**Figure 5:**
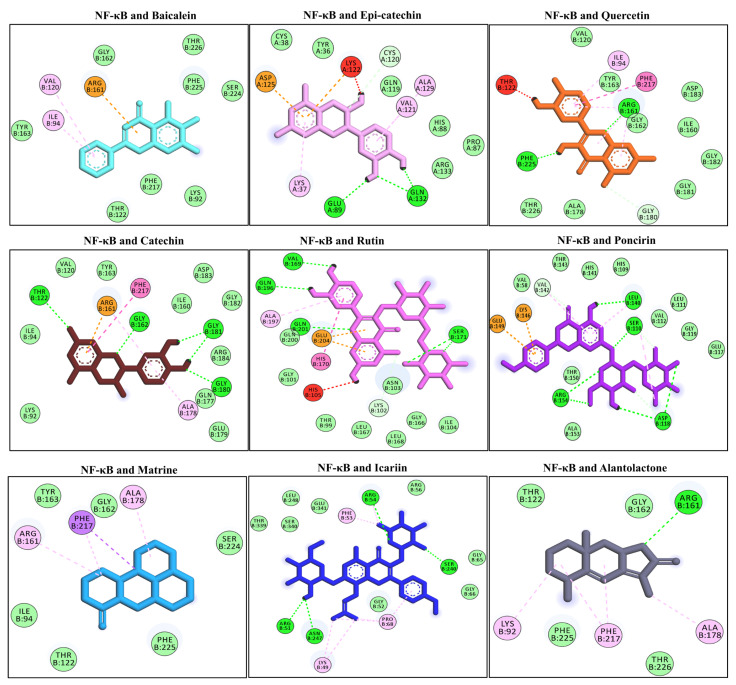
The molecular docking analysis of the selected compounds with the NF-κB receptor using AutoDock Vina software. The molecular docking showed binding of the studied ligand with the NF-κB via multiple hydrophilic and hydrophobic bonds. The ligand and protein interactions were visualized in Discovery Studio Visualizer_16 and ligands whose negative binding energy was high were plotted.

NF-κB activation is followed by interaction of the LPS with the TLR-4 receptor. NF-κB translocates to the nucleus to induce the pro-inflammatory cytokine genes and aggravates the underlying inflammation [48]. Several compounds have shown promising binding affinities with NF-κB via multiple hydrophilic and hydrophobic bonds. The compounds that showed the highest negative binding energies included alantolactone, catechin, epi-catechin, icariin, poncirin, quercetin, and rutin. Alantolactone binds with one H-bond (Ard B161), catechin via four H-bonds (Thr B122, Gly B162, Gly B181, Gly B180), epi-catechin via two H-bonds (Glu A89, Gln A132), icariin via four H-bonds with the NF-κB (Arg B54, Ser B240, Asn B247, Arg B51), poncirin with four H-bonds (Leu B140, Ser B110, Asp B118, Arg B154), quercetin via two H-bonds (Arg B161, Phe B225), and rutin showed four H-bonds with NF-κB (Val B169, Gln B196, Gln B201, Ser B171), as shown in Figure 5. The molecular docking energies are represented in kcal/mol and are presented in Table 5.

### 3.9. Docking Interaction with the MAPKs (JNK)

**Figure 6:**
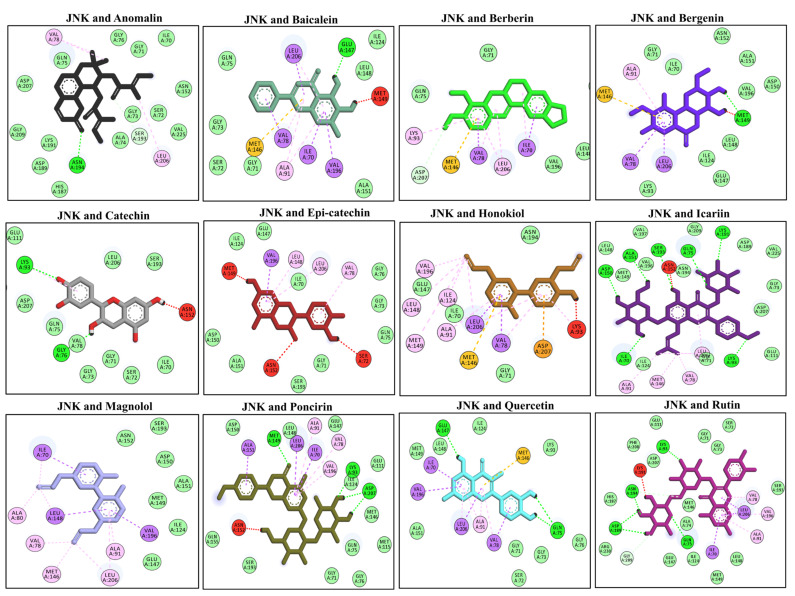
The molecular docking analysis of the selected compounds with the JNK receptor using AutoDock Vina software. The molecular docking showed binding of the studied ligand with the JNK via multiple hydrophilic and hydrophobic bonds. The ligand and protein interactions were visualized in Discovery Studio Visualizer_16 and ligands whose negative binding energy was high were plotted.

The molecular docking interaction was performed targeting the mitogen activating protein kinases (MAPKs) [49]. The MAPK activation is under the influence of upstream signaling proteins such as MyD88, TIRAF, and TRAP [50]. The phosphorylation of MAPKs triggers the activation of the downstream transcription factor (i.e., AP-1). The phosphorylated JNK interacts with the downstream transcription factor and induces the expression of concerned genes. The molecular docking analysis showed the highest negative binding energies with the anomalin, alnatolactone, baicalein, berberin, bergenin, catechin, epi-catechin, honokiol, icariin, magnolol, poncirin, quercetin, and rutin. Anomalin binds with one H-bond (Asn A194), baicalein (Glu A147), bergenin (Met A149), catechin bind with one H-bond (Lys A93), icariin binds with seven H-bonds (Asp A150, Ala A151, Lys A191, Lys A93, Ile A70, Ser A193, Gln A75), poncirin via three H-bonds (Met A 149, Lys A93, Asp A207), quercetin via two H-bonds (Gln A75, Glu A147), and rutin via four H-bonds with the JNK protein (Lys A93, Asn A194, Asp A189, Gln A75), as showbn in Figure 6. The molecular docking energies are represented in kcal/mol and are presented in Table 5.

### 3.10. Docking Interaction with the AP-1

**Figure 7:**
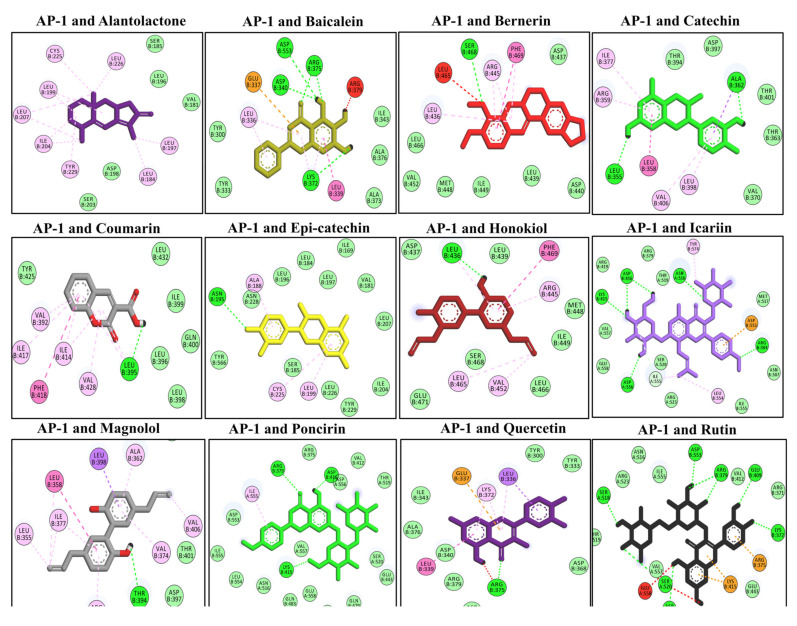
The molecular docking analysis of the selected compounds with the AP-1 protein using AutoDock Vina software. The molecular docking showed binding of the studied ligand with AP-1 via multiple hydrophilic and hydrophobic bonds. The ligand and protein interactions were visualized in Discovery Studio Visualizer_16 and ligands whose negative binding energy was high were plotted.

The computational analysis was performed to assess the binding interaction and explore the possible mechanism of interaction of the studied compounds with the AP-1 transcriptional factor [49]. AP-1 is a transcriptional factor that is activated when it receives upstream signals from the MAPK protein. Following activation, AP-1 induces the expression of various genes associated with inflammation and oxidative stress [51]. The molecular docking analysis revealed the highest negative binding energies for several selected compounds such as alanatolactone, baicalein, bergenin, catechin, continentalic acid, coumarin, epi-catechin, honokiol, icariin, magnolol, poncirin, quercetin, and rutin. Baicalein binds with AP-1 via four H-bonds (Asp B553, Arg B375, Asp B340, Lys B372), bergenin via one H-bond (Ser B468), catechin via two H-bonds (Ala B362, Leu B355), coumarin via one H-bond (Leu B395), honokiol (Leu B436), icariin bind with five H-bonds (Asn A516, Asp B416, Lys B415, Arg B304, Asp A556), magnolol binds with one H-bond (Thr B394), poncirin with three H-bonds (Arg B379, Asp B416, Lys B415), quercetin via a single H-bond (Arg B375), and rutin via seven H-bonds (Ser A518, Asp B533, Arg B379, Glu B409, Lys B372, Ser A520, Asp B556), as shown in Figure 7. The molecular docking energies are represented in kcal/mol and are presented in Table 5.

### 3.11. Analysis of MD Simulation and Calculation of Secondary Structure Content

The MD simulation studies were performed to assess the binding stability of TLR-4 and rutin using YASARA software [52]. The root mean square fluctuation and root mean square displacement were measured to assess the stability of the TLR-4 and rutin complex [52]. The results of the MD simulation were obtained from the TLR-4 protein backbone flexibility by plotting the RMSD, RMSF, and radius of gyration of all of the amino acid residues of TLR-4 [52].

### 3.12. RMSD Analysis

**Figure 8:**
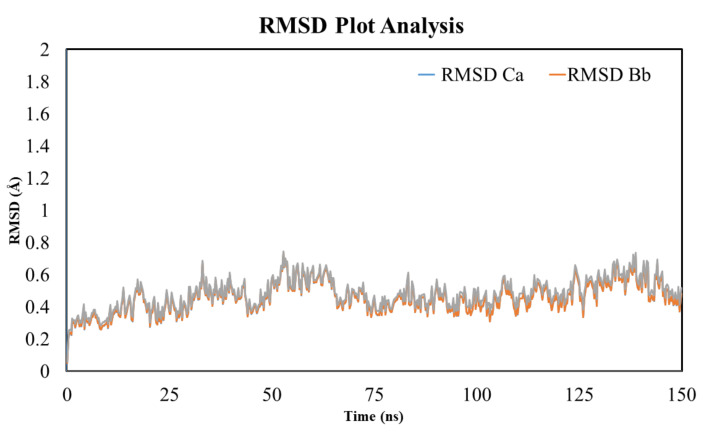
The MD simulation analysis using the root mean square deviation (RMSD) plot. The MD simulation was performed to assess the RMSD value of the TLR-4 and rutin complex for 150 ns. The MD simulation analysis showed a RMSD score within the acceptable range and no significant deviation was observed.

The stability of the ligand (i.e., rutin within the active pocket of the TLR-4 receptor) and the effect of the ligand on the stability of the overall ligand–protein complex was assessed using the RMSD plot. The results of the simulation revealed that the complex was stable and the RMSD was within the acceptable region (less than 2 Å). The average RMSD value was found to be in the range of 0.2 and 0.75. Figure 8 shows that the complex remained stable; however, at a few points during the MD simulation, there was some fluctuation for a small period within the acceptable range (avg. RMSD of 0.45 Å). Afterward, the complex became stabilized for the rest of the simulation (i.e., 150 ns). Thus, the result of the simulation suggests a stable internal motion and minimum fluctuation during the whole process of simulation. The difference in RMSD is associated with the binding and unbinding of the ligand with the protein with the passage of time. Furthermore, the stability of the system can be assessed from the smooth entrance of the system into the production phase and the ligand remains intact with the protein. Apart from this, the small molecule simulation affected the system in a different way when the orientation of the ligand changed over time during the MD simulation.

### 3.13. RMSF Analysis

**Figure 9:**
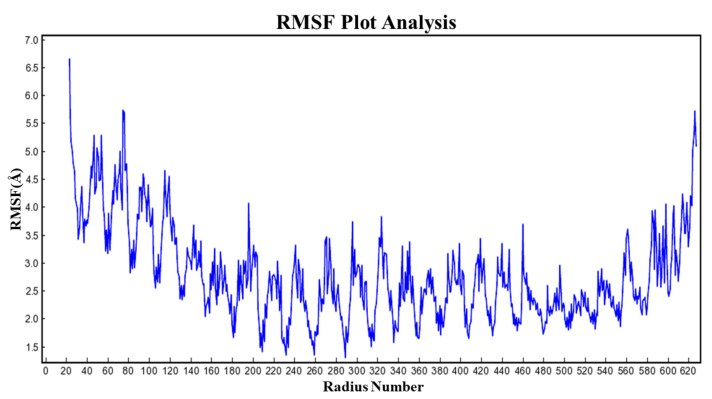
The MD simulation analysis using the root mean fluctuation (RMSF) plot. The molecular dynamic simulation revealed that the RMSF value of the TLR-4 and rutin complex was within the acceptable range and no significant deviation was noted.

The RMSF analysis exhibited the flexibility of the residues. As evident from Figure 9, the flexibility pattern was almost similar and was noted between 4.05 to 4.25 in all secondary components, except for the region where the loop was located and showed an increased fluctuation of the residues, as shown in Figure 9. However, the residue of the active site remains stable during the process of simulation because of the active site recognition of the ligand. The findings of the simulation showed that TLR-4 was stabilized following binding of the rutin ligand (i.e., the binding of the ligand markedly affected the fluctuation of the residue) and is due to the internal residue’s disturbance by the ligand interaction with TLR-4, which influences the correlated and non-correlated motions.

### 3.14. Radius of Gyration

**Figure 10:**
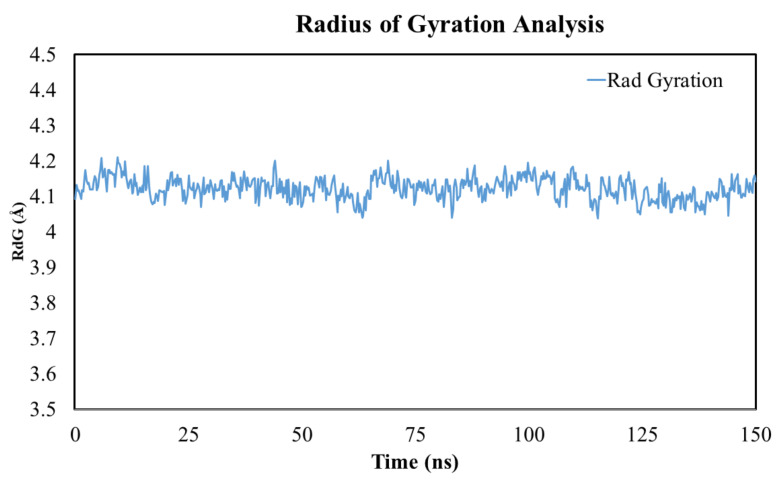
The MD simulation analysis using the radius of gyration (RdG) plot. The MD simulation analysis of the TLR-4 and rutin complex showed a radius of gyration within the acceptable range.

The radius of gyration (RoG) provides insights into the stability of the folding and unfolding of protein during ligand interaction within the protein. The high compactness (more folding) indicates a low Rdg value and higher structural stiffness, while low compactness (more unfolding) indicates a high RdG and less structural stiffness. In short, the RdG value tends to provide information about the system compactness during simulation. As evident from Figure 10, the ligand–protein complex showed a gyration score of 1.5 to 5.5 Å across the whole duration of the simulation. Relative high fluctuation at the C- and N-terminal of the protein was due to large-scale conformational motions. Based on the MD simulation data, we can decipher the overall compactness of the complexes, which is greatly influenced by the binding and unbinding of the ligand within the protein.

## 4. Discussion

The inflammatory response is considered to be a protective mechanism to fend off the offending agent, however, if not dealt with properly, it can lead to deleterious effects on the body [53]. The inflammatory process is associated with the plethora of events such as vascular dilation, immune cells infiltration, and the production of pro-inflammatory mediators [54]. During inflammatory insult, the vascular compartment becomes dilated and facilitates the infiltration of the immune cells. The infiltrated immune cells aim to counter the offensive agents and inhibit the systemic dissemination of the inflammatory response [54]. The infiltrated immune cells trigger the release of pro-inflammatory mediators such as cytokines, chemokines, and even free radicals [55]. These mediators further facilitate the infiltration of the immune cells and inflammatory mediators, which is associated with the tissue damage and discomfort. Several efforts are underway to explore new avenues for the treatment of inflammation and associated symptoms [56]. Plants are a rich and cheap source of drug development, and numerous plant-derived products are in clinical practice for the ailment of various diseases [57]. Computational drug design has recently been of focus for new drug development. In recent times, its use for the discovery of new drug development and the screening of large libraries of compounds in a very short time has tremendously increased [56].

The current study investigated the natural compounds for anti-inflammatory activities targeting TLR-4/AP-1 signaling [58]. TLR-4 signaling plays a crucial role in inflammation following binding with the LPS, which induces the downstream pathway to stimulate the production of inflammatory cytokines [8]. The TLR-4 activation recruits the cytosolic protein such as MyD88 and TIRAP toward the plasma membrane, which crosstalk with the NF-κB and MAPKs (JNK). The NF-κB remains inactive within the cytoplasmic due to the inhibitory action of the IκB and cannot translocate into the nucleus to augment the production of pro-inflammatory genes [8]. However, during stressful conditions, IκB undergoes phosphorylation and degradation, which leads to the activation of NF-κB. Once, the NF-κB (p65 unit) enter the nucleus, it induces the expression of concerned genes associated with inflammation such as cytokines. Similarly, the MAPK protein is downstream to MyD88 and is responsible for the induction of pro-inflammatory cytokines via AP-1 signaling. The AP-1 serves as transcriptional factors and associated with the regulation of numerous genes [58].

The molecular docking analysis of the selected compound with TLR-4 showed a difference in the binding energies and the docking score ranged between −4.7 kcal/mol with pimelic acid and −10.4 kcal/mol energy with rutin. These ligands showed multiple hydrophilic and hydrophilic binding with the protein targets. According to the study of Weitao Fu et al. (2016), it was found that inhibiting the TLR-4 receptor caused downstream signaling to be inhibited, resulting in a significant reduction in the inflammatory response in several in vitro and in vivo investigations. Furthermore, Li-Shuang Hou et al. (2020) showed that the inhibition of the TLR-4 receptor by rutin improved the hepatic inflammation and reduced the hepatic fibrosis [59]. Furthermore, inhibiting TLR-4 upstream caused a decrease in the expression of downstream signaling proteins including MAPKs and NF-κB, which resulted in a reduction in inflammatory cytokines [59]. Additionally, the molecular docking analysis of the selected compounds with the MAPKs (JNK) revealed multiple interactions via both hydrophilic and hydrophobic bonds. However, the docking score of the JNK with the selected compounds ranged between −4.6 kcal/mol and −9.5 kcal/mol. Pimelic acid showed the lowest binding energy (−4.6 kcal/mol) and poncirin exhibited the highest binding energy (−9.5 kcal/mol). Poncirin interacted with the JNK protein by multiple H-bonds and hydrophobic bonds. The JNK is linked downstream with the AP-1 (activated protein-1) transcriptional factor, which induces the expression of multiple genes concerned with inflammation, and thus by inhibiting the JNK protein, it will lead to the reduced expression of pro-inflammatory genes [60]. The result of the present study is consistent with the study of Ullah et al. (2022), which reported that the inhibition of the JNK significantly reduced the hepatic inflammation and inflammatory mediators [60]. Similarly, the molecular docking analysis showed the lowest binding energy of pimelic acid with the AP-1 protein (−5.4 kcal/mol) and the highest binding energy with icariin (−9.1 kcal/mol). Icariin showed multiple H-bonds and hydrophobic bonds with the AP-1 protein. The AP-1 comprised of two subunits (i.e., c-fos and c-jun )and its activation by the MAPK upstream protein significantly induced its activation and aggravated the inflammatory response. The result of the present study was supported by the study of Hosek et al. (2019), where the inhibition of the AP-1 signaling significantly reduced the inflammatory cytokines and pain associated with the rheumatoid arthritis [61].

Furthermore, the molecular docking with tNF-κB showed binding energies between −4.9 kcal/mol (linoleic acid) and −9.4 kcal/mol (poncirin). The NF-κB and selected compounds showed multiple hydrophilic bonds and hydrophilic bonds. Poncirin showed the highest binding affinity for the NF-κB and interacted via multiple H-bonds and hydrophobic bonds. The NF-κB, once activated following degradation of the IkB within the cytosol, was translocated to the nucleus and induced the expression of the genes concerned with inflammatory cytokines such as IL-1B, IL-6, and TNF-a. The result of the present study was supported by Khan et al. (2019) where the increased expression of the IkB protein inhibited the NF-κB activation, which led to significant improvement in inflammation and inflammatory arthritis [1].

The computational approach has gained significant attention in the rapid screening of physico-chemical properties of new drugs [62]. The computational methods provide an important insight into the molecular weight, LogP value, number of rotational bonds, number of acceptors and donors, and surface area. The computational analysis showed the diverse physico-chemical behavior of the selected compounds. The various toxicity tests that were evaluated includes carcinogenicity, cardiotoxicity using herG enzyme, hepatotoxicity, skin sensitivity, minnow toxicity, and maximum tolerated dose. The maximum compounds showed no carcinogenicity, most of the compounds exhibited no toxicity against the liver, and no minnow toxicity was observed. Prior to introducing a substance into clinical practice, it is critical to investigate its toxicity on essential organs such as the liver, kidneys, and heart [63,64]. Similarly, detailed information on the carcinogenicity and mutagenicity is critical during pre-clinical trials to avoid the harmful effects of such substances on humans. Information on the chemical toxicity assessment is not only crucial, but also very time-consuming by employing in vitro and in vivo methods [63,64]. As a result, computer-based assessment can help examine the toxicokinetic aspects of a large number of compounds in a short amount of time [65]. Similarly, a computer-based toxicity assessment, which includes carcinogenicity, mutagenicity, and influence on vital organ toxicities, is increasingly widely used in pre-clinical investigations since it reduces the toxicity evaluation time and has excellent reliability, as previously reported [63,64]. The pharmacokinetic parameters of the selected compounds were assessed using computational approaches [55]. Any compound’s pharmacokinetic characteristic has a major impact on the compound’s pharmacodynamic activity [21]. Absorption, bioavailability, volume of distribution, hydrophilicity, lipophilicity, permeability into the brain, metabolism, and excretion are all aspects of pharmacokinetics [21,60]. Using in vitro and in vivo models to determine the pharmacokinetics of a large number of drugs is time consuming, expensive, and challenging [21,60]. In silico pharmacokinetic investigations, on the other hand, are an efficient way of screening a large number of drugs in a short amount of time, are inexpensive and have a high level of reliability. The pharmacokinetic parameters were assessed using in silico analysis in this study [60,65]. The various parameters that were assessed included intestinal absorption, the blood–brain barrier, Caco-2 permeability, cytochrome p450, and P-glycoprotein activity. The 14 compounds showed high GIT absorption, 10 compounds also showed BBB properties, and the selected compounds portrayed differences in their glycoprotein and cytochrome p450 activities. Similarly, the drug-likeness properties were found for all of the selected compounds using SwissADME software. The drug-likeness properties showed that most of the compounds obeyed the Lipinski rule, Ghose, Veber, Egan, and Muegge. The boiled egg analysis was performed using SwissADME software to assess the BBB, human intestinal absorption, and P-glycoprotein substrate. Several selected compounds showed BBB permeability and P-glycoprotein substrate activity.

Computational analysis is a fast and efficient method of screening compounds for a number of disorders including inflammation [66,67]. TLR-4 signaling leads downstream signaling proteins to become active during inflammatory conditions, boosting the expression of pro-inflammatory cytokines, and aggravating the underlying inflammatory disease. By targeting the upstream TLR-4 and downstream signaling cascades, the compounds were investigated for their inflammatory activities using molecular docking, MD modeling, and ADMET analysis [67]. This study sheds light on the selected compounds’ potential anti-inflammatory and anti-arthritic efficacy against the molecular signaling pathways implicated in inflammatory arthritis. The compounds with the highest binding energy can be employed to treat rheumatoid arthritis following further in-depth analysis. Rheumatoid arthritis affects a large percentage of the population, and its cases are steadily rising, having a substantial impact on the patient’s quality of life. The various treatment options are unable to effectively reduce all of the symptoms associated with the numerous interactable side effects. As a result, there is a need for alternative rheumatoid arthritis treatments that are both effective and safe. Because several compounds demonstrated promising activity against the inflammatory target, no significant toxicity against key organs, and acceptable pharmacokinetic features, this study provides important insights into the treatment of rheumatoid arthritis.

The current study used molecular docking, MD simulation, and ADMET analysis to provide a detail computational investigation of the selected compounds. The computational analysis revealed the potential binding energies for various compounds and revealed the inflammatory protein target’s maximum affinity, while the MD analysis revealed the stability of the ligand–protein complex. The ADMET study showed a wide range of pharmacokinetic and toxicokinetic features for the selected compounds. The compounds were not examined after computational analysis using in vitro and in vivo analysis, which is a major limitation of the study. The computational analysis can be verified by conducting detailed in vitro and in vivo analyses against the studied target, particularly for those molecules with the highest binding energy [67]. Similarly, the compound with the highest binding energy was not studied utilizing an animal model or a cell-based assay for pharmacokinetic and toxicokinetic studies.

## 5. Conclusions

The molecular docking analysis showed different binding energies of the selected compounds with the TLR-4, NF-κB, JNK, and AP-1 proteins. The compounds exhibited binding with the protein’s targets via multiple hydrophilic and hydrophobic bonds. Furthermore, the MD analysis showed significant stability of the ligand–protein complex, as evident from the RMSD, RMSF, and RoG. Furthermore, these compounds also portrayed diverse physico-chemical, drug-likeness, pharmacokinetic, and toxicokinetic properties. Several compounds have exhibited strong affinities for TLR-/AP-1 signaling, which can be employed for the treatment of inflammatory disorders. The study’s key limitation is that anti-inflammatory and anti-arthritic efficacy in vitro and in vivo were not examined for the compounds with promising binding energies. The chemicals were also not tested in an in vivo model, despite substantial in silico pharmacokinetic and toxicokinetic analysis.

## Figures and Tables

**Table 1:** The analysis of the physico-chemical properties of the selected compounds using computational analysis.

S. No	Molecular Weight	LogP	Rotatable Bonds	Acceptors	Donors	Surface Area
Anomalin	426.465	4.3925	4	7	0	179.832
Alantolactone	232.323	3.2406	0	2	0	102.727
Baicalein	270.24	2.5768	1	5	3	112.519
Berberin	336.367	3.0963	2	4	0	144.867
Bergenin	328.273	−1.2006	2	9	5	129.813
Catechin	290.271	1.5461	1	6	5	119.662
Continentalic acid	302.458	5.2062	2	1	1	134.232
Coumarin	146.145	1.793	0	2	0	63.079
Epi-catechin	290.271	1.5461	1	6	5	119.662
Ferulic acid	194.186	1.4986	3	3	2	81.065
Gallic acid	170.12	0.5016	1	4	4	67.135
Honokiol	266.34	4.2218	5	2	2	118.887
Icariin	676.668	0.0679	9	15	8	273.926
Linoleic acid	28.452	5.8845	14	1	1	124.520
Magnolol	266.34	4.2218	5	2	2	118.887
Matrine	248.37	1.8717	0	2	0	109.506
P-coumaric acid	164.16	1.49	2	2	2	69.587
Pimelic acid	160.169	1.1061	6	2	2	64.840
Poncirin	594.566	−0.8622	7	14	7	239.713
Quercetin	302.238	1.988	1	7	5	122.108
Rutin	610.521	−1.6871	6	16	10	240.901
Vanillic acid	168.148	1.099	2	3	2	69.025

**Table 2:** The toxicity assessment of the selected compounds using computational assessment.

S. No	Ames Toxicity	herG I Inhibitor	herG II Inhibitor	Hepatotoxicity	Skin Sensitization	T. Pyriformis Toxicity	Minnow Toxicity	Max. Tolerated Dose (Human)	Oral Rat Acute Toxicity (LD50)	Oral Rat Chronic Toxicity (LOAEL)
Anomalin	NO	NO	NO	YES	NO	0.365	0.325	0.135	3.266	2.137
Alantolactone	NO	NO	NO	NO	YES	1.237	0.936	0.042	1.597	1.856
Baicalein	NO	NO	NO	NO	NO	0.42	1.25	0.498	2.325	2.645
Berberin	YES	NO	NO	YES	NO	0.354	−0.277	0.144	2.571	1.89
Bergenin	NO	NO	NO	NO	NO	0.285	5.688	−0.013	1.879	3.614
Catechin	NO	NO	NO	NO	NO	0.347	3.585	0.438	2.428	2.5
Continentalic acid	NO	NO	NO	YES	NO	0.31	−0.35	0.008	1.838	2.207
Coumarin	NO	NO	NO	NO	NO	0.365	1.555	0.435	2.112	1.903
Epi-catechin	NO	NO	NO	NO	NO	0.347	3.585	0.438	2.428	2.5
Ferulic acid	NO	NO	NO	NO	NO	0.271	1.825	1.082	2.282	2.065
Gallic acid	NO	NO	NO	NO	NO	0.285	3.188	0.7	2.218	3.06
Honokiol	NO	NO	YES	NO	NO	0.749	0.14	0.305	2.184	1.791
Icariin	NO	NO	YES	NO	NO	0.285	5.51	0.451	2.631	5.081
Linoleic acid	NO	NO	NO	YES	YES	0.701	−1.31	−0.827	1.429	3.187
Magnolol	NO	NO	YES	NO	NO	0.941	−0.054	0.468	1.976	1.851
Matrine	NO	NO	NO	NO	YES	0.56	2.264	0.141	2.54	0.874
P-cumaric acid	NO	NO	NO	NO	NO	0.319	1.607	1.111	2.155	2.534
Pimelic acid	NO	NO	NO	NO	NO	−0.103	2.006	0.106	1.338	3.11
Poncirin	NO	NO	YES	NO	NO	0.285	5.65	0.259	2.545	4.096
Quercetin	NO	NO	NO	NO	NO	0.288	3.721	0.499	2.471	2.612
Rutin	NO	NO	YES	NO	NO	0.452	2.491	3.673	0.285	7.677
Vanillic acid	NO	NO	NO	NO	NO	0.265	1.926	0719	2.454	2.032

**Table 3:** The pharmacokinetic parameter assessment of the selected compounds using the computational approach.

Compounds	GI Absorption	BBB Permeation	Caco-2 Permeability	P-Glycoprotein Substrate	P-Glycoprotein Inhibitor	CYP1A2 Inhibitor	CYP2C19 Inhibitor	CYP2C9 Inhibitor	CYP3A4 Inhibitor
Anomalin	High	NO	0.938	NO	YES	NO	YES	YES	YES
Alantolactone	High	YES	1.603	NO	YES	NO	YES	YES	NO
Baicalein	High	NO	1.117	NO	NO	YES	NO	NO	YES
Berberin	High	YES	1.734	YES	NO	YES	NO	NO	YES
Bergenin	Low	NO	0.289	YES	NO	NO	NO	NO	NO
Catechin	Low	NO	−0.283	YES	NO	NO	NO	NO	NO
Continentalic acid	High	YES	1.742	NO	NO	NO	YES	YES	NO
Coumarin	High	YES	1.649	NO	NO	YES	NO	NO	NO
Epi-catechin	Low	NO	−0.283	YES	NO	NO	NO	NO	NO
Ferulic acid	High	YES	0.176	NO	NO	NO	NO	NO	NO
Gallic acid	Low	NO	−0.081	NO	NO	NO	NO	NO	YES
Honokiol	High	YES	1.586	YES	NO	YES	YES	YES	YES
Icariin	Low	NO	−0805	YES	YES	NO	NO	NO	NO
Linoleic acid	High	YES	1.57	NO	NO	YES	NO	YES	NO
Magnolol	High	YES	1.707	YES	NO	YES	YES	YES	YES
Matrine	High	YES	1.463	YES	NO	NO	NO	NO	NO
P-cumaric acid	High	YES	1.21	NO	NO	NO	NO	NO	NO
Pimelic acid	High	NO	0.598	NO	NO	NO	NO	NO	NO
Poncirin	Low	NO	0.62	YES	YES	NO	NO	NO	NO
Quercetin	Low	NO	−0.229	YES	NO	YES	NO	NO	YES
Rutin	Low	NO	−0.949	YES	NO	NO	NO	NO	NO
Vanillic acid	High	NO	0.33	NO	NO	NO	NO	NO	NO

**Table 4:** The drug-likeness behavior of the selected compounds using computational analysis.

Compounds	Lipinski	Ghose	Veber	Egan	Muegge
**Anomalin**	YES	YES	YES	YES	YES
**Alantolactone**	YES	YES	YES	YES	YES
**Baicalein**	YES	YES	YES	YES	YES
**Berberin**	YES	YES	YES	YES	YES
**Bergenin**	YES	NO	NO	NO	YES
**Catechin**	YES	YES	YES	YES	YES
**Continentalic acid**	YES	YES	YES	YES	NO
**Coumarin**	YES	NO	YES	YES	NO
**Epi-catechin**	YES	YES	YES	YES	YES
**Ferulic acid**	YES	YES	YES	YES	NO
**Gallic acid**	YES	NO	YES	YES	NO
**Honokiol**	YES	YES	YES	YES	YES
**Icariin**	NO	NO	NO	NO	NO
**Linoleic acid**	YES	NO	NO	NO	NO
**Magnolol**	YES	YES	YES	YES	YES
**Matrine**	YES	YES	YES	YES	YES
**P-cumaric acid**	YES	YES	YES	YES	NO
**Pimelic acid**	YES	NO	YES	YES	NO
**Poncirin**	NO	NO	NO	NO	NO
**Quercetin**	YES	YES	YES	YES	YES
**Rutin**	NO	NO	NO	NO	NO
**Vanillic acid**	YES	YES	YES	YES	NO

**Table 5:** The molecular docking score of the selected compounds against the TLR-4, JNK, NF-κB, and AP-1 proteins.

Sample	TLR-4 (4g8e) Energy (kcal/mol)	JNK (30xi) Energy (kcal/mol)	P65 (1le5) Energy (kcal/mol)	AP-1 (4hmy) Energy (kcal/mol)
Anomalin	−7.0	−7.0	−6.6	−7.0
Alantalactone	−6.2	−7.1	−7.1	−8.7
Baicalein	−7.7	−8.1	−8.0	−8.6
Berberin	−7.6	−8.2	−6.9	−8.1
Bergenin	−7.5	−7.5	−6.7	−6.9
Catechin	−7.5	−7.3	−7.3	−7.9
Continentalic acid	−7.3	−6.8	−6.9	−7.5
Coumarin	−6.2	−6.9	−6.3	−7.9
Epi-catechin	−7.9	−7.6	−7.6	−8.6
Ferulic acid	−5.9	−6.2	−5.7	−6.9
Gallic acid	−6.0	−5.6	−6.0	−6.1
Honokiol	−6.7	−7.2	−7.1	−7.8
Icariin	−9.1	−9.4	−7.0	−9.1
Linoleic acid	−4.7	−5.9	−4.9	−5.9
Magnolol	−6.9	−7.5	−6.9	−8.9
Matrine	−6.1	−6.8	−7.1	−6.6
P-coumaric acid	−5.8	−6.1	−5.2	−6.6
Pimelic acid	−4.5	−4.6	−5.3	−5.4
Poncirin	−10.1	−9.5	−9.4	−8.0
Quercetin	−8.0	−7.8	−7.3	−8.0
Rutin	−10.4	−9.1	−7.8	−8.6
Vanillic acid	−6.1	−5.6	−5.2	−6.2

## Data Availability

The original contributions presented in the study are included in the article; further inquiries can be directed to the corresponding authors.

## Sample Availability

Samples of the compounds are available from the authors.
